# Hydrothermal derived nitrogen doped SrTiO_3_ for efficient visible light driven photocatalytic reduction of chromium(VI)

**DOI:** 10.1186/s40064-016-2804-2

**Published:** 2016-07-19

**Authors:** Guanjie Xing, Lanxiao Zhao, Tao Sun, Yiguo Su, Xiaojing Wang

**Affiliations:** College of Chemistry and Chemical Engineering, Inner Mongolia University, Hohhot, Inner Mongolia 010021 People’s Republic of China

**Keywords:** Doping, Photocatalysis, Chromium(VI) reduction, Strontium titanate

## Abstract

In this work, we report on the synthesis of nitrogen doped SrTiO_3_ nanoparticles with efficient visible light driven photocatalytic activity toward Cr(VI) by the solvothermal method. The samples are carefully characterized by X-ray diffraction, scanning electron microscopy, transmission electron microscopy, UV–Vis diffuse reflectance spectroscopy and photocatalytic test. It is found that nitrogen doping in SrTiO_3_ lattice led to an apparent lattice expansion, particle size reduction as well as subsequent increase of Brunner–Emmet–Teller surface area. The visible light absorption edge and intensity can be modulated by nitrogen doping content, which absorption edge extends to about 600 nm. Moreover, nitrogen doping can not only modulate the visible light absorption feature, but also have consequence on the enhancement of charge separation efficiency, which can promote the photocatalytic activity. With well controlled particle size, Brunner–Emmet–Teller surface area, and electronic structure via nitrogen doping, the photocatalytic performance toward Cr(VI) reduction of nitrogen doped SrTiO_3_ was optimized at initial hexamethylenetetramine content of 2.

**Electronic supplementary material:**

The online version of this article (doi:10.1186/s40064-016-2804-2) contains supplementary material, which is available to authorized users.

## Background

Hexavalent chromium (Cr(VI)) is a common pollutant detected in groundwater originated from excessively released of electroplating, pigment production and tanning of leather, etc. (Wang et al. 2013a). Cr(VI) has raised considerable attention because of its high toxic, intense mobility and strong teratogenic activity to human organisms. The World Health Organization (WHO) has stipulated that Cr(VI) concentration in drinking water should be below 0.05 ppm (Chen et al. 2011). Precipitation (Gheju and Balcu 2011), adsorption (Sun et al. 2014a, b), ion exchange (Edebali and Pehlivan 2010) and membrane separation (Hsu et al. 2013) as conventional techniques are commonly used to eliminate Cr(VI) from wastewater. Precipitation and adsorption processes are economic and effective, but only efficient when the Cr(VI) concentration is relatively high (Abyaneh and Fazaelipoor 2016; Hokkanen et al. 2016). Ion exchange is high-efficiency in general, but it is rather expensive to maintain and operate (Ali et al. 2015). Some even cause secondary pollution. For example, solvent extraction method could bring in organic pollutants, sulfide precipitator as common precipitator may be residual and generate hydrogen sulfide (H_2_S). In general, conventional techniques are either low efficiency or cost too much when they are applied to low Cr(VI) concentration in wastewater (Wang et al. 2012a; Huang and Huang 1996).

Semiconductor photocatalytic reduction technology has attracted a lot of attention in recent years (Miseki et al. 2008; Kato and Kudo 2002; Wang et al. 2009; Mu et al. 2011; Nakhjavan et al. 2012; Duo et al. 2015; Zhang et al. 2009). Semiconductor photocatalytic technology has a promising prospect for wastewater Cr(VI) removing because it is efficiency and inexpensive to maintain and operate without secondary pollution (Hu et al. 2014; Meichtry et al. 2014; Gherbi et al. 2013; Alanis et al. 2013). Strontium titanate (SrTiO_3_) could be applied in Cr(VI) ion contaminant reduction with excellent photocatalyst performance, but it is only effective under ultraviolet irradiation which is about 4 % of the sunlight (Zheng et al. 2011). That is to say, strontium titanate is ineffective under visible light irradiation when applied to photocatalysis because it has a 3.2 eV band gap energy (Dong et al. 2012). Many leading groups also take advantage of the high bandgap. Li Ji group and Ib Chorkendorff group use SrTiO_3_ and TiO_2_ as protective window layers for Si photocathode during water splitting, and they achieve good result (Ji et al. 2015; Bae et al. 2016). Doping with nonmetal atoms to SrTiO_3_ material could hoist the valence band edge and extend its optical absorption edge towards the visible light range, resulting in visible light driven photocatalytic activity (Sulaeman et al. 2011; Zou et al. 2012). The perovskite phases materials characteristics depend on the anionic composition to a large extent. Therefore, replacing oxygen with other anions, take nitrogen for example, can greatly influence the physicochemical property of the material. There are many reports about doping action including anionic dopant species and metals ions, and anionic doping could narrow the desired semiconductor band gap better than cation ions doping (Khan et al. 2002; Chen and Burda 2008).

In our work, nitrogen-doped SrTiO_3_ powders are synthesized by hydrothermal method reaction. We take hexamethylenetetramine as doping sources and KOH as mineralizer to obtain the fine particles with excellent photocatalytic activity. The nitrogen doping effects on SrTiO_3_ nanoparticles are fully studied in an attempt to investigate the microstructure, optical properties and the relevance to the improved photocatalytic activity toward chromium(VI) reduction.

## Experimental section

### Synthesis of nitrogen doped SrTiO_3_ samples

Titanium tetraisopropoxide Ti(OC_3_H_7_)_4_ and strontium nitrate Sr(NO_3_)_2_·4H_2_O were used as starting materials, hexamethylenetetramine (HMT) as nitrogen source, and KOH as mineralizer. All of them were reagent grade and used without further purification. SrTiO_3_ was prepared by hydrothermal method. Ti(OC_3_H_7_)_4_ was dissolved in 10 mL 2-propanol firstly, Sr(NO_3_)_2_ aqueous solution was added to Ti(OC_3_H_7_)_4_ propanol solution dropwise with continuously stirring. Then, 0–8 g of HMT and 20 mL of 2 M KOH aqueous solution were added to the suspension in turn. The solution was placed into a Teflon container with a stainless steel autoclave outside and then the solution was heated at 200 °C for 3 h in an oven. After that, the autoclave was cooled to room temperature naturally, the obtained powder was washed with distilled water and alcohol three times and dried in vacuum at 60 °C overnight (Sulaeman et al. 2010). The final samples were labeled as pure SrTiO_3_, N-SrTiO_3_(0.5), N-SrTiO_3_(1), N-SrTiO_3_(2), N-SrTiO_3_(3), N-SrTiO_3_(4), N-SrTiO_3_(5), N-SrTiO_3_(6) and N-SrTiO_3_(8) with the increased HMT content.

### Sample characterization

X-ray power diffraction (XRD) was applied to characterize the purity and crystallinity of all our samples (D8 Advance Bruker X-ray diffractometer, CuKα radiation, 2θ = 20–80^o^). Transmission electron microscopy (TEM) was used to determine the morphology of the as-prepared samples (JEM-2010 apparatus, 200 kVA acceleration voltage). Diffusive reflectance UV–Vis spectrophotometer (Perkin-Elmer Lambda35) was employed to measure the samples UV–Vis absorption, BaSO_4_ was taken as the reference sample. Barrett–Emmett–Teller (BET) technique was taken to determine the specific surface areas (Micromeritics ASAP 2000 Surface Area and Porosity Analyzer). X-ray photo spectrometer (XPS) analysis was employed for sample element state (ESCALab220i-XL). PGSTAT302 N potentiostat galvanostat Autolab electrochemical working station using a standard three-compartment cell was used for photoelectrochemical characteristics under 300 W Xe arc lamp (≥420 nm). The fluorine-doped tin oxide (FTO) glasses (0.6 cm^2^) were washed for 30 min using absolute ethanol with ultrasonication. 0.1 g sample mixed with 0.01 g Polyvinylidene fluoride (PVDF) and 0.5 mL N-methyl pyrrolidinone (NMP) were placed in an glass bottle under magnetic stirring for at least 8 h. Then the obtained mixtures were coated on the FTO glasses. Photocatalyst solution was coated onto the FTO glasses substrate by drop casting using 5 μL pipette tip, and 3 drops were enough. Then we use the pipette tip to smooth the film at room temperature in the air. Lastly, the coated FTO glasses were dried for 4 h at 60 °C in the air. Photocatalyst coated FTO glass, a piece of Pt sheet, an Ag/AgCl electrode and 0.5 M sodium sulfate were used as the working electrode, counter electrode, reference electrode and electrolyte, respectively.

### Photocatalytic reactivity test

Disposal ability of Cr(VI) was evaluated through photo-reduction under 300 W mercury lamp with a filter (λ ≥ 400 nm) irradiation in photochemical reactor. 50 mL 5 mg L^−1^ Cr(VI) solution with pH adjusted to ≈1 containing 30 mg photocatalyst was magnetically stirred during photocatalytic test. 4 mL suspension was sampled at selected intervals and centrifuged (10,000 rpm, 5 min). The content of Cr(VI) was determined using diphenylcarbazide colorimetrical method (Ma et al. 2014). The reduction efficiency of Cr(VI) was calculated according to the formula:

$$\eta = ({\text{C}}_{0} - {\text{C}}_{t} )/{\text{C}}_{0} \times 100\;\%$$

where η is the photocatalytic efficiency, C_0_ is the initial concentration of Cr(VI), and C_t_ is the concentration of Cr(VI) after illumination (for time t).

## Results and discussion

**Fig. 1:**
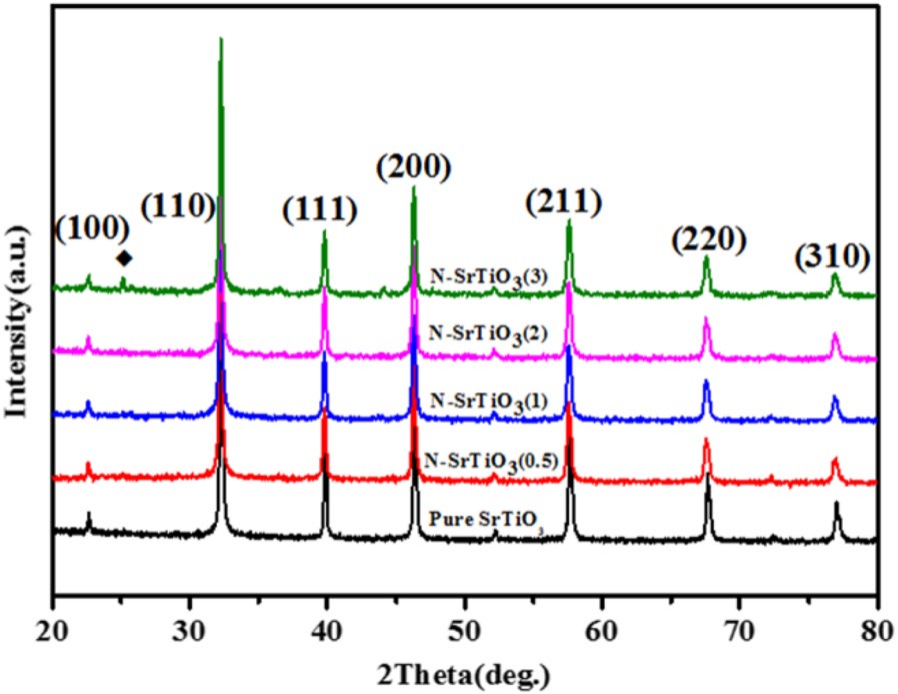
XRD patterns of pure SrTiO_3_ and nitrogen doped SrTiO_3_ with different initial HMT content

Figure 1 showed the XRD patterns of the as-prepared samples. It was clearly seen that all diffraction peaks of pure SrTiO_3_ could be readily indexed to a well-crystallized perovskite phase of SrTiO_3_ (JCPDS no. 01-074-1296). There were no peaks belonging to other phases, indicating SrTiO_3_ was phase pure. As for the nitrogen doped SrTiO_3_ samples, it was observed that the diffraction peaks were identical to that of the pure SrTiO_3_ except for the weakened intensity and narrowed full width at half maximum (FWHM) of the corresponding peaks with the increasing initial HMT content, suggesting a particle size reduction may occurred by the nitrogen doping action. It was noted that further increase of HMT content to 3.0 g led to the appearance of additional diffraction peaks belonging to SrCO_3_(2θ = 25.15°, 25.79°) as shown in Fig. 1 and Additional file 1: Fig. S1. Structure refinement of the as prepared samples was carried out using general structure analysis system (GSAS) software (Larson and Von Dreele 1994). Rietveld refinement patterns of pristine SrTiO_3_ and N-SrTiO_3_ (2) samples were shown in Additional file 1: Fig. S2(a) and (b). The lattice parameters of pure SrTiO_3_ and N-SrTiO_3_(2) samples were listed in Additional file 1: Table S1. It was noted that the lattice volume of N-SrTiO_3_ sample expanded from 59.676 to 60.043 Å^3^. Several factors should be taken into consideration to clarify this phenomenon, including particle size, doping effect, surface feature, and so forth. Lattice expansion as a function of particle size reduction is not new and has been well documented in many semiconductors (Wang et al. 2013b; Mikhailovskaya et al. 2013). This can be ascribed to the surface structural relaxation due to the abundant surface exposed atoms. For the present SrTiO_3_ samples, the primary particle size was estimated to be ~48 and 39 nm for pure SrTiO_3_ and N-SrTiO_3_(2) samples, respectively, from the diffraction peak (110) using Scherrer formula, indicating much large particle size, which was also verified by TEM observations. Basically, large particle size implies less surface exposed atoms, which leads to negligible variation of lattice parameters. On the other hand, doping effects will not only alter the lattice parameters and electronic structure, but also have consequence on the physical properties (Wang et al. 2013b; Mikhailovskaya et al. 2013). Nitrogen tends to occupy at the oxygen site in SrTiO_3_ host matrix and results in lattice expansion because (1) the ionic radius of N^3−^ is 0.146 nm, which is a bit larger than 0.138 nm of O^2−^, (2) Pauling’s electronegativity of nitrogen is 3.04, which is close to that of 3.44 for oxygen. This conception can be further verified by XPS observations.

**Fig. 2:**
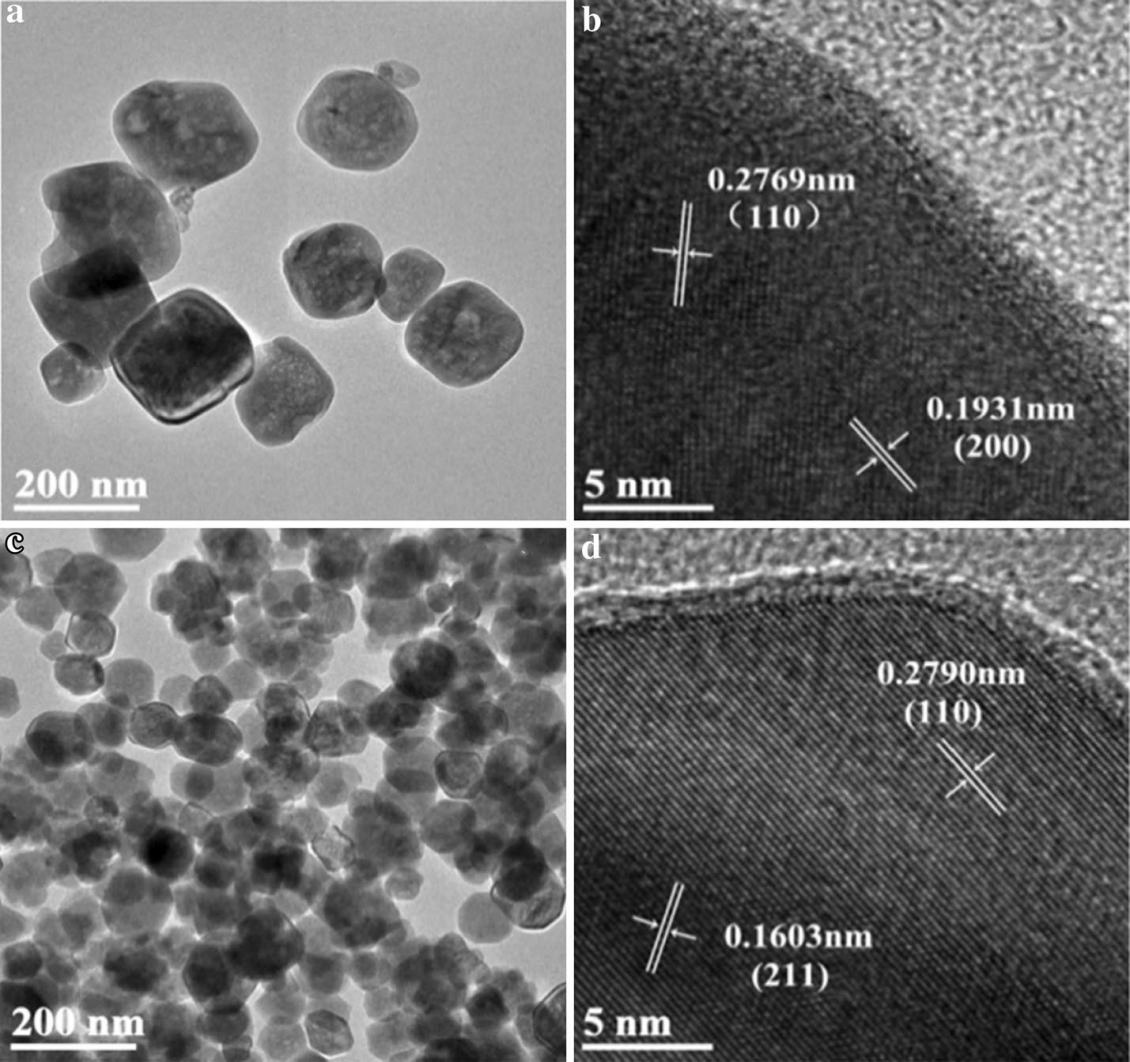
TEM (**a**) and HRTEM (**b**) images of pure SrTiO_3_, TEM (**c**) and HRTEM (**d**) images of N-SrTiO_3_(2)

Transmission electron microscopy (TEM) was employed for samples morphology and crystal structure. Figure 2a showed the TEM image of pure SrTiO_3_ nanoparticles. It can be seen that pure SrTiO_3_ exhibited irregular cubic-like morphology with an average diameter of about 200 nm, which is larger than that obtained from XRD results. High resolution TEM (HRTEM) image indicated that pure SrTiO_3_ exhibited high crystalline feature with the space between the adjacent of 0.2769 and 0.1931 nm, which was close to 0.276 and 0.194 nm for (110) and (200) plane of SrTiO_3_, respectively (Fig. 2b). As for nitrogen doped samples, the diameter of N-SrTiO_3_(2) showed a particle size reduction to ~100 nm with the same cubic-like morphology (Fig. 2c). HRTEM image for N-SrTiO_3_(2) was shown in Fig. 2d. As illustrated in Fig. 2d, the lattice plane space was determined to be 0.2790 and 0.1603 nm, which is compatible to (110) and (211) plane for SrTiO_3_.

**Fig. 3:**
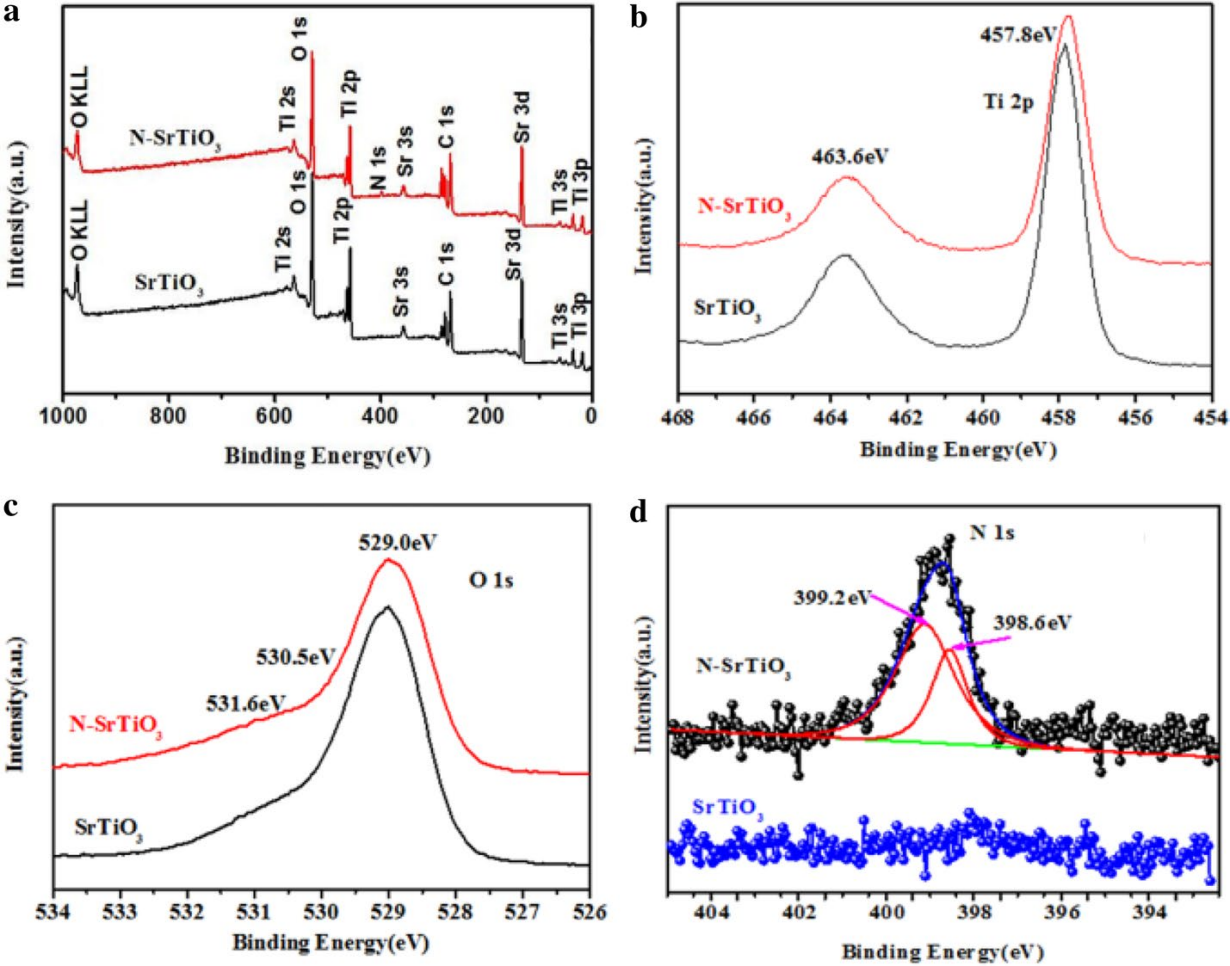
XPS spectra of pure and nitrogen doped SrTiO_3_ samples: the whole scanning spectra (**a**) and the high resolution regional spectra of Ti 2p (**b**), O 1 s (**c**), and N 1 s (**d**)

XPS technique was carried out to specify the chemical composition and charge state of the samples. Figure 3a shows the XPS global spectra of pure SrTiO_3_ and N-SrTiO_3_(2). From Fig. 3a, it was seen that all peaks can be ascribed to Sr, Ti, O, and C elements for pure SrTiO_3_ and Sr, Ti, O, N and C for nitrogen doped SrTiO_3_. Figure 3b showed the high resolution XPS spectra of Ti 2p for pure and nitrogen doped SrTiO_3_. The binding energies of Ti 2p_3/2_ and Ti 2p_1/2_ appeared at about 457.6 and 463.4 eV, respectively, which is nearly identical to the typical values of SrTiO_3_ (Sulaeman et al. 2011). Apparently, as shown in Fig. 3b, an about ~0.3 eV red shift was observed after the incorporation of nitrogen. This observation is related to the fact that an increase of the electron density on Ti occurred because the electronegativity of the N atom is smaller than the O atom (Wang et al. 2012b). The O 1 s peak in the XPS spectra of pure SrTiO_3_ and nitrogen doped SrTiO_3_ was shown in Fig. 3c. The peaks appeared at 529.0, 530.5 and 531.6 eV, which can be ascribed to lattice oxygen atoms, surface hydroxyl groups and surface chemisorbed oxygen, respectively (Su et al. 2015). The N-species in the N-SrTiO_3_ were displayed at 398.8 eV of N1 s XPS peak (Fig. 3d), which can be fitted into two peaks. The peak observed at binding energy of 399.2 eV was ascribed to sample surface adsorbed nitrogen while peak at 398.6 eV could be assigned to the shift of Ti–N bonds. The shift was very likely to be induced by the formation of O_vac_ and the Ti^3+^ cations adjacent to the vacancies (Zou et al. 2012).

**Fig. 4:**
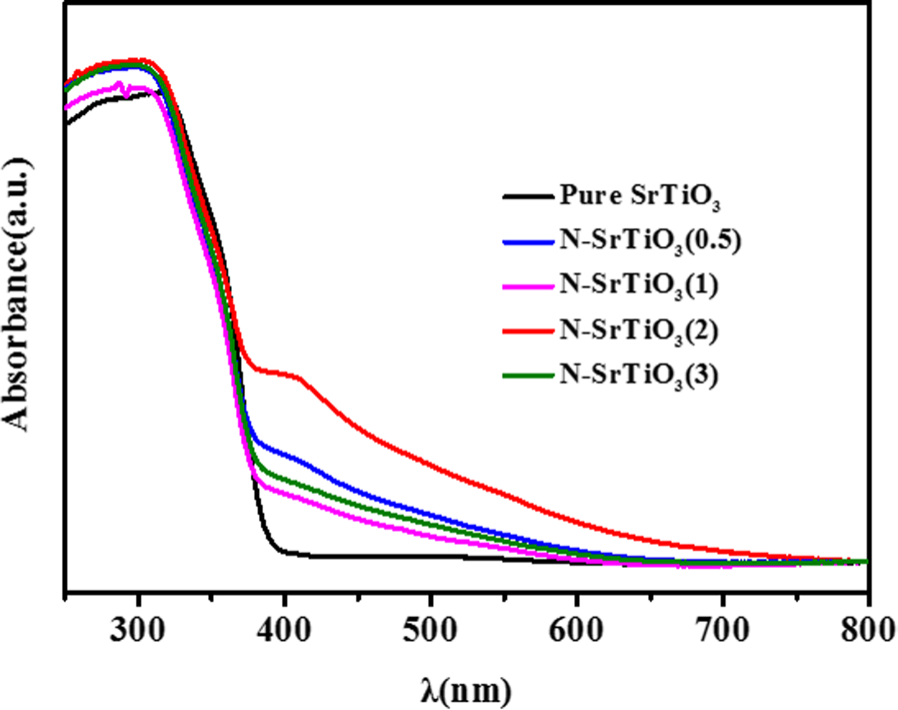
UV–visible diffuse reflection spectra of pure SrTiO_3_ and nitrogen doped SrTiO_3_ samples

The UV–Vis absorption performance of the samples was employed to investigate the electronic transitions and band gap energies of nitrogen doped SrTiO_3_. As shown in Fig. 4, absorption edge of pure SrTiO_3_ was about 386 nm, and the band gap energy was estimated to be 3.21 eV. Furthermore, a new absorption band at about 400–550 nm was observed in N-doped SrTiO_3_ samples which may be resulted from N 2p to Ti 3d orbital electron transition. Interestingly, the absorption intensity of N-doped SrTiO_3_ gradually increased with an increase of the initial HMT content. Moreover, the absorption edge also broadened to the higher wavelength more than 550 nm. It is presumed that the absorption above 550 nm is due to oxygen vacancy. When doping nitrogen into SrTiO_3_, there was possibility that O^2−^ was replaced by N^3−^ which would result in anion defects for the charge compensation of O^2−^ and N^3−^ (Zou et al. 2012). Therefore, the formation of the new band gaps in the visible light region could most possibly be ascribed to anion defects which were formed by nitrogen doping.

**Fig. 5:**
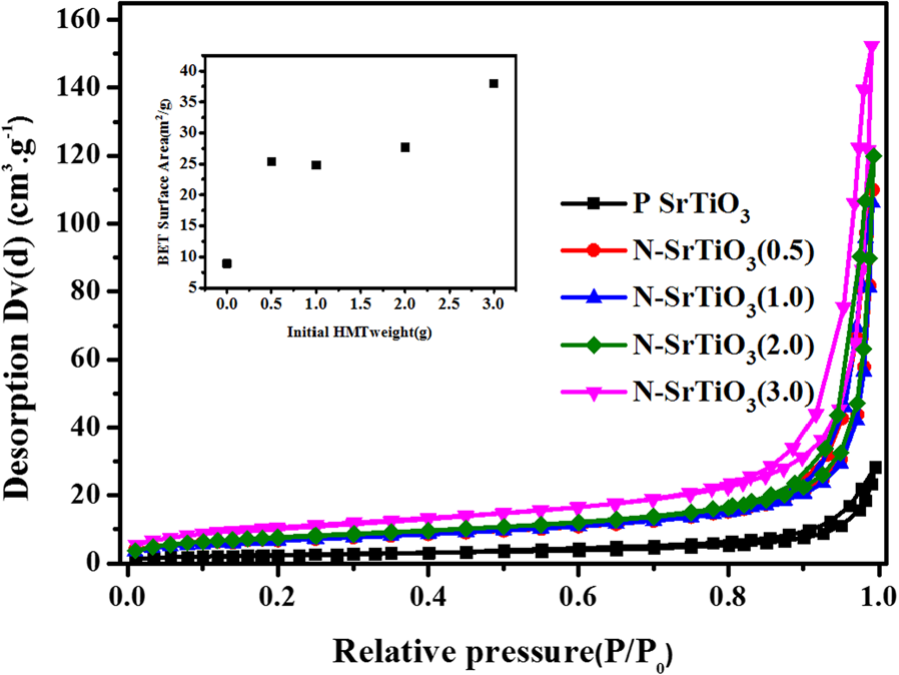
Nitrogen adsorption-desorption isotherms of pure SrTiO_3_ and nitrogen doped samples. The inset shows the BET surface area as a function of initial HMT content

BET measurement was adopted to detect the impacts of nitrogen doping on surface areas of SrTiO_3_. As shown in Fig. 5, the isotherm curves of SrTiO_3_ samples with different nitrogen content were characterized by a hybrid type between type I and IV in the Brunauer Deming-Dming-Teller (BDDT) classification. It is worth noting that the BET surface area of pure SrTiO_3_ nanoparticles is to be about 8.89 m^2^/g, which, however, increases to ~37.94 m^2^/g by nitrogen doping (inset of Fig. 5). The increase of surface area of the nitrogen doped SrTiO_3_ nanoparticles may predict greatly enhanced photocatalytic activities.

**Fig. 6:**
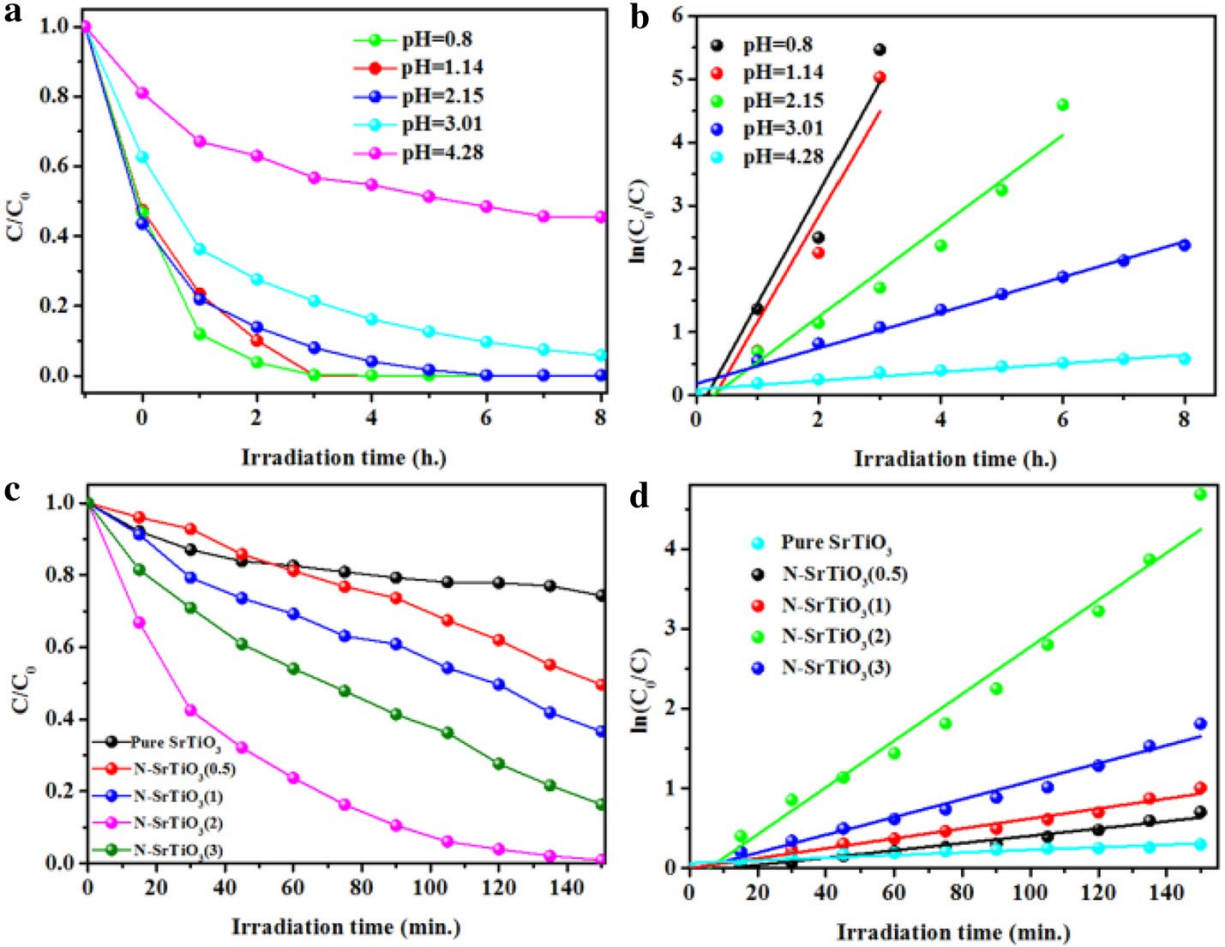
Influence of initial pH on the Cr(VI) removal effect (**a**) and relationship between ln(C_0_/C) and the reaction time (**b**). Cr(VI) concentration as a function of visible light irradiation time in the presence of nitrogen doped SrTiO_3_ with various initial HMT content (**c**) and relationship between ln(C_0_/C) and the reaction time (**d**)

Cr(VI) reduction was used as the model reaction to investigate the doping effects of nitrogen on the photocatalytic performance of SrTiO_3_. Cr(VI) suspensions were magnetically stirred to ensure the establishment of an adsorption/desorption equilibrium before illumination. Little Cr(VI) was adsorbed for all the as-prepared samples. Cr(VI) concentration was monitored using the standard diphenylcarbazide method with maximal adsorption at ~540 nm. The solution pH plays an important role on the reduction of Cr(VI). A series of experiments was conducted by varying solution pH from 0.5 to 4.3 to investigate pH influence. The pH influence of N-SrTiO_3_(2) sample was presented in Fig. 6a. It was clear that the photocatalytic efficiency increased by degrees when adjust the initial pH from 4.28 to 0.5. The lower of the pH led to the higher of the photocatalytic efficiency. On the basis of other group’s report, the relevant reactions during the reduction of Cr(VI) were as follows (Sun et al. 2014c; Mu et al. 2010):

$$\begin{aligned} &{\text{N{-}SrTiO}}_{3} \mathop{\longrightarrow}\limits^{h\upsilon }e^{ - } + h^{ + } \hfill \\ &{\text{Cr}}_{2} {\text{O}}_{7}^{2 - } + 14{\text{H}}^{ + } + 6e^{ - } \to 2{\text{Cr}}^{3 + } + 7{\text{H}}_{ 2} {\text{O}} \hfill \\ & 2{\text{H}}_{2} {\text{O}} + 4h^{ + } \to {\text{O}}_{2} + 4{\text{H}}^{ + } \hfill \\ \end{aligned}$$

**Fig. 7:**
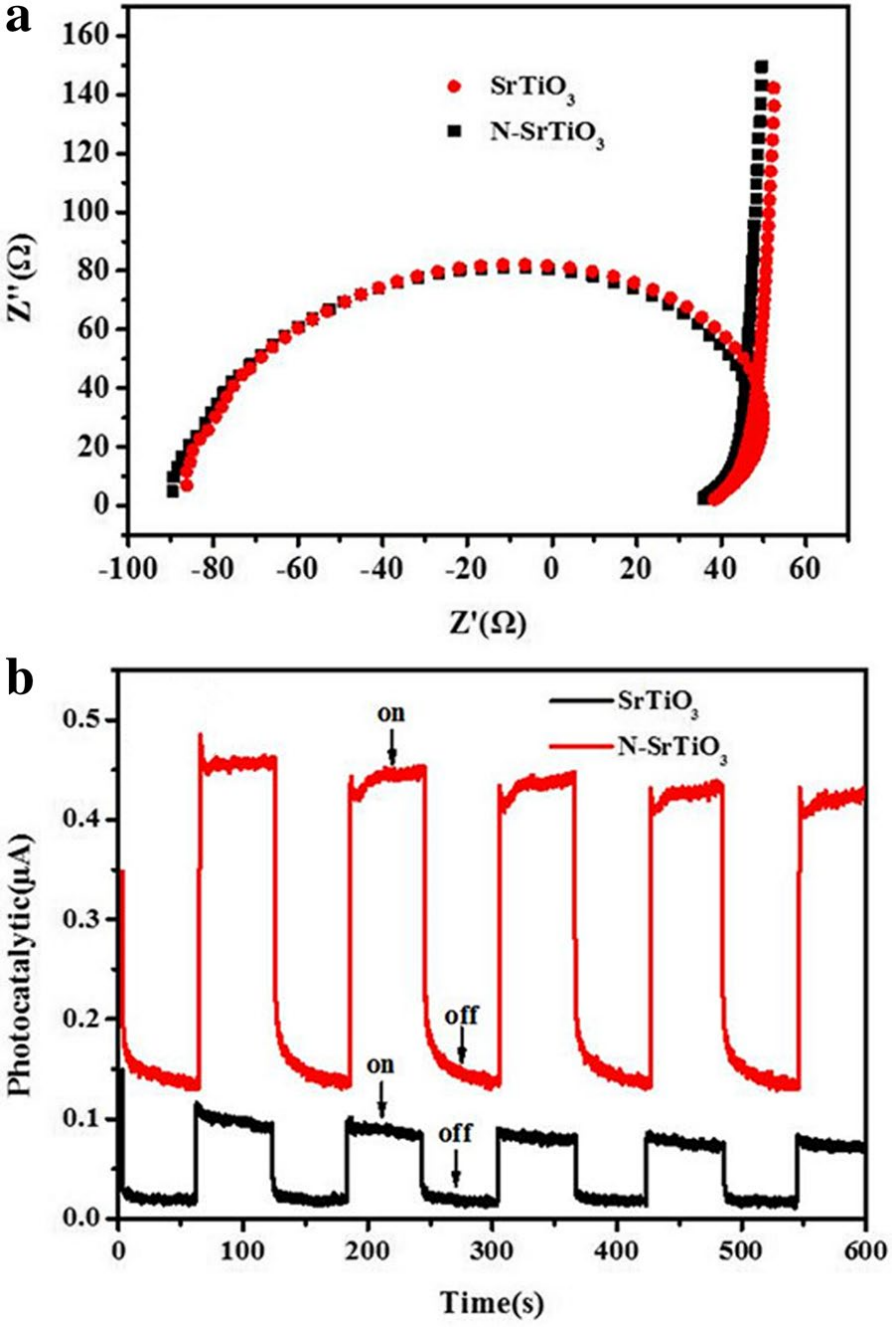
Electrochemical impedance spectroscopy (EIS) plots (**a**) and photocurrent responses of the SrTiO_3_ and N-SrTiO_3_(2) electrodes (**b**)

It was seen that the higher H^+^ concentration predicted higher reduction activity of Cr(VI) over nitrogen doped SrTiO_3_. Thus, the photocatalytic reduction efficiency of Cr(VI) can be promoted at a lower pH condition. Considering the harm of low pH value to the facilities in application, we adopted pH≈1 in the later Cr(VI) reduction test. It is noted that there is a linear relationship between ln(C_0_/C) and reaction time as seen in Fig. 6b, indicating the reduction of Cr(VI) proceeds through a pseudo-first-order kinetic reaction, ln(C_0_/C) = k_app_t, where k_app_ is the apparent rate constant. The optimal apparent rate constant was determined to be 1.37 h^−1^ at pH value of 1. As seen in Fig. 6c, the photocatalytic performance of N-SrTiO_3_ can be flexibly tuned by the initial HMT content. With the increase of the initial nitrogen content from 0 to 2 g, N-SrTiO_3_ showed increasing photocatalytic activity and N-SrTiO_3_(2) revealed the optimized photocatalytic activity. The reduction efficiency of 5 mg/L^−1^ Cr(VI) achieved to about 100 % after about 2 h under visible light irradiation. However, as for N-SrTiO_3_(3) sample, there was a decrease in the photocatalytic performance. Moreover, the reduction of Cr(VI) proceeds through a pseudo-first-order kinetic reaction over all nitrogen doped SrTiO_3_ samples as depicted in Fig. 6d. Two synergistic effects may be the reasons for the photocatalytic performance of the N-SrTiO_3_ samples. One was that nitrogen doping action affected the surface feature of SrTiO_3_ nanoparticles strongly. Doping action resulted in an increase of the specific surface area of the nitrogen doped SrTiO_3_ samples which provided a better access for the Cr(VI) molecules to approach the as-prepared samples and then the Cr(VI) reduction reaction can easily proceed. Another factor is related to the band gap narrowing and efficient charge separation which was resulted from both N-dopants and the O_vac_. Because of the O_vac_, conduction band minimum (CBM) of nitrogen doped SrTiO_3_ shifted more positive. At the same time, valence band maximum of nitrogen doped SrTiO_3_ moved more negative for the reason of N-dopants (Zou et al. 2012). The efficient charge separation in nitrogen doped SrTiO_3_ can be verified by Nyquist plot and photocurrent measurements. As shown in Fig. 7a, the observed arcs for both pure and nitrogen doped SrTiO_3_ samples were depressed which was often observed in solid electrolytes impedance spectra. The arc radius on the Nyquist plot of N-SrTiO_3_ electrode was almost the same with that of SrTiO_3_ electrode. Generally speaking, photocurrent value could give an index to the sample’s ability to generate and transfer the photogenerated charge carriers indirectly, which impact samples photocatalytic performance to a big degree (Dong et al. 2014). The photocurrent plots of the SrTiO_3_ and N-SrTiO_3_ electrodes under visible light was shown in Fig. 7b which was obtained at a bias voltage of 0.5 V. SrTiO_3_ sample showed a photocurrent of 8.85 × 10^−8^ A while an enhanced photocurrent of 3.98 × 10^−7^ A for N-SrTiO_3_ sample was observed at the same condition. The photocurrent for N-SrTiO_3_ was nearly 4.5 times as much as the SrTiO_3_ sample which indicated that the faster charge transfer and more effective separation of electron-hole pairs through the N-SrTiO_3_ electrode interface, which can drastically enhance the photocatalytic activity toward Cr(VI) reduction.

**Fig. 8:**
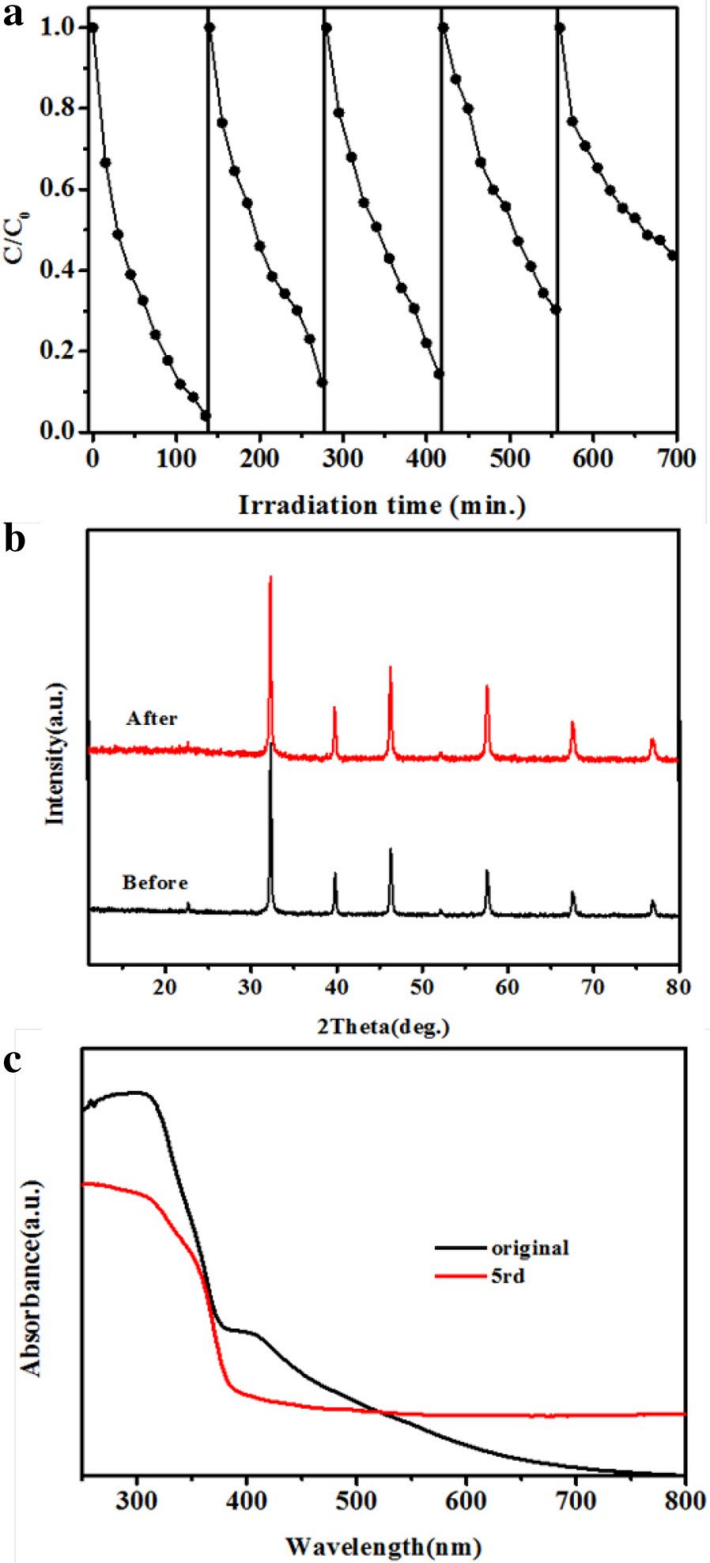
Cycling performance of N-SrTiO_3_(2) toward Cr(VI) reduction (**a**), XRD patterns of N-SrTiO_3_(2) after 5 cycles recycle test toward Cr(VI) reduction (**b**), UV–visible diffuse reflection spectra of of N-SrTiO_3_(2) after 5 cycles recycle test toward Cr(VI) reduction (**c**)

To check the stability of the N-SrTiO_3_(2) photocatalyst, recycle test was carried out. The XRD pattern and UV–visible diffuse reflection spectra of N-SrTiO_3_(2) after the reduction of Cr(VI) was measured, and was shown in Fig. 8b, c. The XRD patterns peak of the N-SrTiO_3_(2) sample did not change much before and after the Cr(VI) reduction test, suggesting that our photocatalyst was stable in structure. But as seen in Fig. 8c, visible light absorption decreased much after 5rd recycle test which may result from nitrogen loss in the nitrogen doped SrTiO_3_, being often observed in several nitrogen doped oxides (Kumar et al. 2014; Ruzimuradov et al. 2015). This may also be the reason for decrease of photocatalytic performance in Fig. 8a as 55 % of the photocatalytic activity was preserved after 5 cycles. In summary, nitrogen doped SrTiO_3_ possess excellent photocatalytic performance towards Cr(VI) reduction under visible light, but the stability of the nitrogen doped SrTiO_3_ sample still needs to be further improved.

## Conclusions

In summary, nitrogen doped SrTiO_3_ nanoparticles with controlled particle size, electronic structure and efficient visible light driven photocatalytic activity toward Cr(VI) were successfully prepared by a solvothermal method. XRD, BET and TEM analyses indicated that nitrogen doped SrTiO_3_ nanoparticles with cube-like morphology exhibited an apparent lattice expansion, particle size reduction as well as subsequent increase of BET surface area via nitrogen doping. The visible light absorption edge and intensity can be modulated by nitrogen doping content, which absorption edge extends to about 600 nm. Moreover, nitrogen doping can not only modulate the visible light absorption feature, but also have consequence on the enhancement of charge separation efficiency, which can promote the photocatalytic activity. With well controlled particle size, BET surface area, and electronic structure via nitrogen doping, the visible light driven photocatalytic performance toward Cr(VI) reduction of nitrogen doped SrTiO_3_ was optimized at initial HMT content of 2. Such a finding may help to provide hints for developing and designing new photocatalytic semiconductors.

## Additional file

10.1186/s40064-016-2804-2 Supplementary data associated with this article.
