# Supplementary material for: Hydrothermal derived nitrogen doped SrTiO3 for efficient visible light driven photocatalytic reduction of chromium(VI)
Source: Springerplus. 2016 Jul 19;5(1):1132. doi: 10.1186/s40064-016-2804-2 (PMC4951392; doi:10.1186/s40064-016-2804-2)
Supplement: Supplementary file 1 — 10.1186/s40064-016-2804-2 Supplementary data associated with this article. [file 40064_2016_2804_MOESM1_ESM.docx]

Supporting Information for

Hydrothermal derived nitrogen doped SrTiO_3_ for efficient visible light driven photocatalytic reduction of chromium(VI)

Guanjie Xing, Lanxiao Zhao, Tao Sun, Yiguo Su^*^, Xiaojing Wang^*^

College of Chemistry and Chemical Engineering, Inner Mongolia University, Hohhot, Inner Mongolia 010021, P. R. China


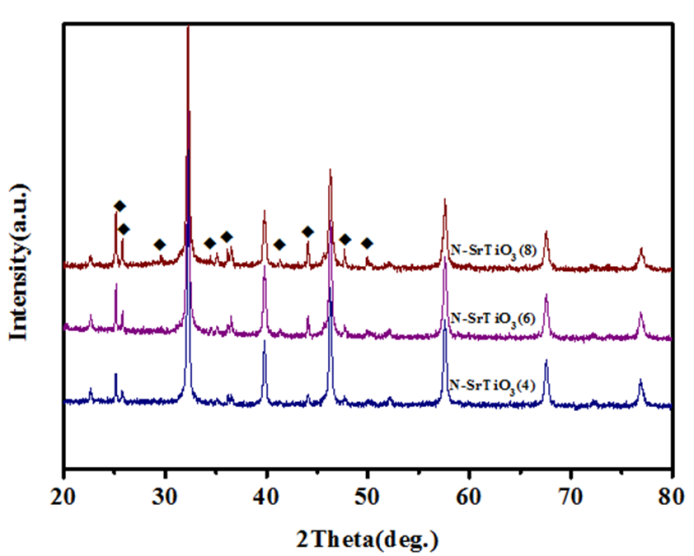


**Fig. S1** XRD patterns of nitrogen doped SrTiO3 with higher initial HMT content.


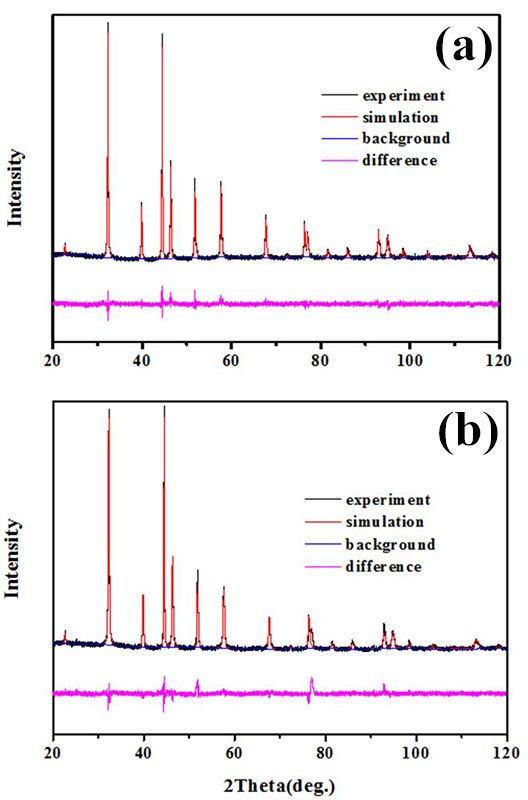


**Fig. S2** Rietveld plot of (a) pure SrTiO_3_, (b) N-doped SrTiO_3_(2).

**Table S1** Lattice parameters of pristine SrTiO_3_ and N-SrTiO_3_(2).

| Initial HMT content | a(Å) | b(Å) | c(Å) | V(Å^3^) |
| --- | --- | --- | --- | --- |
| X=0 | 3.9078 | 3.9078 | 3.9078 | 59.676 |
| X=2 | 3.9158 | 3.9158 | 3.9158 | 60.043 |
